# Diversity of the Fossil Genus *Palaeoglaesum* Wagner (Diptera, Psychodidae) in the Upper Cretaceous Amber of Myanmar

**DOI:** 10.3390/insects12030247

**Published:** 2021-03-16

**Authors:** Kornelia Skibińska, Marzena Albrycht, Qingqing Zhang, Wojciech Giłka, Marta Zakrzewska, Wiesław Krzemiński

**Affiliations:** 1 Institute of Systematics and Evolution of Animals Polish Academy of Sciences, Sławkowska 17, 31-016 Kraków, Poland; wieslawk4@gmail.com; 2 Institute of Biology, Pedagogical University of Kraków, Podchorążych 2, 30-084 Kraków, Poland; marzena.albrycht@up.krakow.pl; 3 State Key Laboratory of Palaeobiology and Stratigraphy, Nanjing Institute of Geology and Palaeontology and Center for Excellence in Life and Paleoenvironment, Chinese Academy of Sciences, 39 East Beijing Road, Nanjing 210008, China; qqzhang@nigpas.ac.cn; 4 Institute of Geosciences, University of Bonn, 53115 Bonn, Germany; 5 Laboratory of Systematic Zoology, Department of Invertebrate Zoology and Parasitology, Faculty of Biology, University of Gdańsk, Wita Stwosza 59, 80-308 Gdańsk, Poland; wojciech.gilka@ug.edu.pl (W.G.); marta.zakrzewska@ug.edu.pl (M.Z.)

**Keywords:** fossil insects, inclusions, Bruchomyiinae, Mesozoic, taxonomy, new species

## Abstract

**Simple Summary:**

Bruchomyiinae is one of seven subfamilies of Psychodidae. In the contemporary fauna, this small, relict subfamily occurs mainly in tropical and sub-tropical regions. Examination of inclusions preserved in the Upper Cretaceous amber of Myanmar (also known as Burmese amber), which is almost 100 mya, shows that this subfamily was abundant during the Cretaceous period. The extinct genus *Palaeoglaesum* is known only from this fossil resin. Moreover, its numerous inclusions and high diversification confirm that the Mesozoic was the stage of the early evolution and radiation of Psychodidae. Here we describe three new species and we provide additional details regarding the morphology of fossil species.

**Abstract:**

Cretaceous Myanmar amber is abundant in inclusions belonging to the genus *Palaeoglaesum*. In addition, a significant morphological diversity among representatives of *Palaeoglaesum* can be observed. However, none of its representatives have been found in other fossil materials. Herein three new species: *P. stebneri* sp. nov., Skibińska and Krzemiński *P. teres* sp. nov. Skibińska and Albrycht, and *P. pilosus* sp. nov. Skibińska, Krzemiński and Zhang from Cretaceous Myanmar amber are described and illustrated. The very small size and characters of male hypopygium with aedeagus strongly bent and apically forked are pertinent to diagnosing the genus and species. New materials show that this genus and the whole subfamily Bruchomyiinae were probably more abundant and more diverse than the presently known extant fauna.

## 1. Introduction

Bruchomyiinae is one of seven subfamilies of Psychodidae (Diptera). It was originally described as a subfamily of Tanyderidae [1], but Edwards [2] indicated that the group is closely related to some extinct genus of the family Psychodidae. Bruchomyiinae comprises 53 extant species (found on all continents excluding Antarctica) which are restricted to tropical and sub-tropical climates [3]. Adults of this group are reported to be mostly associated with forest habitats, especially resting in tree hollows or between buttresses. This is supposed to be the main reason why Bruchomyiinae are relatively abundant in the Myanmar amber [4,5]. The genus *Palaeoglaesum* Wagner, 2017 [6] is known only from the Myanmar amber, and to date this is one of the most common genus of this subfamily (personal observation) in this fossil resin. The known fossil fauna consists of seven species, i.e., *P. velteni* [7]; *P. quadrispiculatus* [8]; *P. mulleri* [6]; *P. bisulcum* [6]; *P. notandum* [6]; *P. carsteni* [5] and *P. wagneri* [5]. A general synopsis of *Palaeoglaesum* was provided by Wagner [6] and later diagnosis of the genus has been revised by Skibińska et al. [5]. This extinct group can be distinguished easily from other bruchomyiines by the noticeably smaller size (about 2–2.5 mm) and generally the body is more robustly setose. This character is usually seen particularly well on legs, which possess a fringe of elongate setae and on the head with a median longitudinal strip of setae, recalling in some species the stereotypical Mohawk hairstyle. Also the characteristic shape of male genitalia with aedeagus elongated, arched toward gonopods and with a bifurcated apex is a distinctive feature of the examined genus.

Herein, we provide a description of three new species belonging to the fossil genus *Palaeoglaesum.* This work implies that the subfamily Bruchomyiinae was far more diverse during the Cretaceous period than was previously thought. Moreover, we intend to provide additional details regarding the morphology of fossil species and try to improve our understanding of the evolution of this genus.

## 2. Materials and Methods

Specimens were examined using a Nikon SMZ25 stereomicroscope (located in ISEA PAS, Kraków, Poland) equipped with a Nikon DS-Ri2 digital camera (located in ISEA PAS, Kraków, Poland). Photomicrographs are focus stacks captured using this system and processed using NIS-Elements Imaging Software (located in ISEA PAS, Kraków, Poland). Line drawings were produced by tracing photographs. Interpretation of wing venation follows Byers [9] and Krzemiński and Krzemińska [10]. General morphological terminology follows Cumming and Wood [11]. The collections in which the specimens are deposited are as follows, with abbreviations used throughout the text: the Natural History Museum of the Institute of Systematics and Evolution of Animals, Polish Academy of Sciences, Kraków, Poland (ISEA PAS); State Key Laboratory of Palaeobiology and Stratigraphy, Nanjing Institute of Geology and Palaeontology, Chinese Academy of Sciences, Nanjing, China (NIGP).

All specimens examined during this study are representatives of Bruchomyiinae preserved as amber inclusion from the Hukawng Valley in Kachin State, northern Myanmar. Burmese amber was dated by Cruickshank and Ko [12] as middle-late Albian based on the index fossil, but Grimaldi et al. [13] estimated the Cenomanian-Turonian age of this resin based on arthropod inclusions. Shi et al., [14] based on U-Pb dating of zircons from the volcaniclastic matrix of the amber, estimated the age of Burmese amber at 98.79 ± 0.62 Ma (earliest Cenomanian).

This published work and the nomenclatural acts it contains have been registered in ZooBank, the online registration system for the International Commission on Zoological Nomenclature (ICZN). The Life Science Identifier (LSID) for this publication is: LSIDurn:lsid:zoobank.org:pub:96AB1938-4F5C-4E8E-A596-8D6A58E44F72.

## 3. Results

### 3.1. Systematic Paleontology

Order Diptera Linnaeus, 1758 [15].

Family Psychodidae Newman, 1834 [16].

Subfamily Bruchomyiinae Alexander, 1920 [1].

Genus *Palaeoglaesum* Wagner, 2017 [6].

Type species: *Nemopalpus quadrispiculatus* Stebner, Solórzano-Kremer, Ibáñez-Bernal and Wagner, 2015: 22, figs. 14 a, b, c, d. Myanmar amber; earliest Cenomanian (Cretaceous).

### 3.2. Description of a New Fossil Material

*Palaeoglaesum stebneri* sp. nov. Skibińska and Krzemiński.

LSID urn:lsid:zoobank.org:act:2982B6E0-A9E8-47EF-AE88-6BD476A495CC.

**Figure 1:**
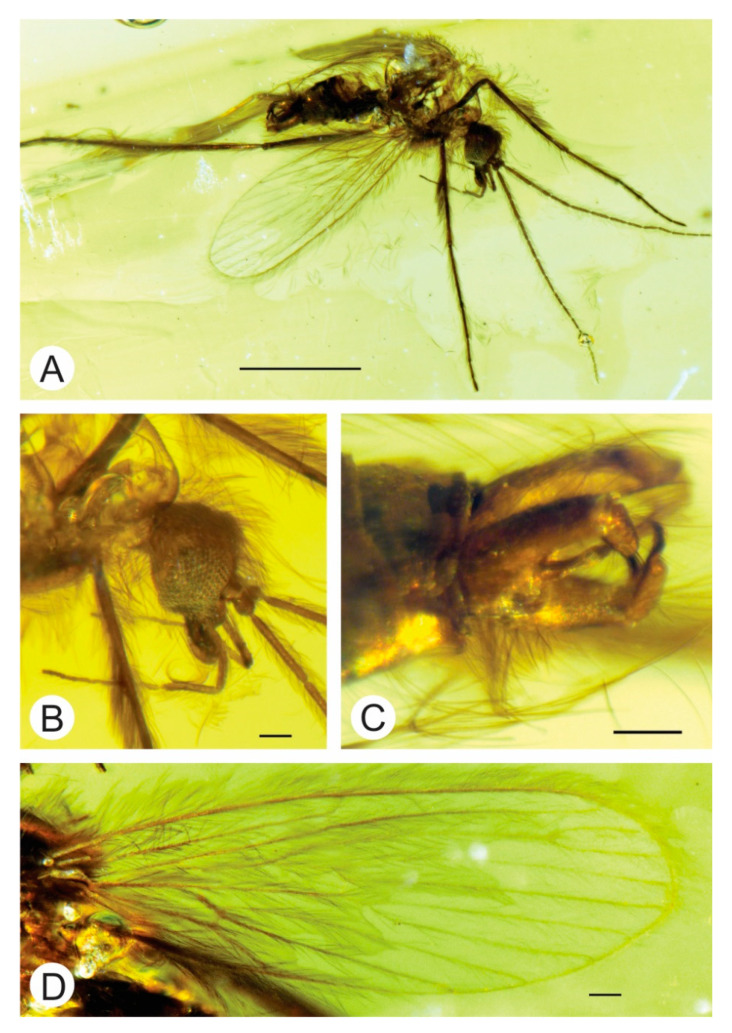
*Palaeoglaesum stebneri* sp. nov. Photos of the holotype [No. MP/4044]. (**A**) Whole specimen, scale: 1 mm; (**B**) head, scale: 0.1 mm; (**C**) male terminalia, scale: 0.1 mm; (**D**) wing, scale: 0.1 mm.

Diagnosis. Male with the following combination of character states: *Wing*: Sc ends almost opposite ¾ of the length of R_4+5_ and slightly before cross-vein r-m; Rs is ¹/₃ as long as R_4+5_ and is equal to the length of R_2_; M_1_ slightly more than 4 times longer than M_1+2_; *Male genitalia*: gonostylus single, straight, with a very strong extension of almost ⅔ of the basic part; the end part of the gonostylus not forked and strongly tapered, slightly sickle-shaped; aedeagus long, slightly curved with apex bifurcate; parameres strongly curved, only slightly shorter than the aedeagus (Figure 1).

Etymology. The new species is named after Frauke Stebner, a specialist working on fossil Diptera, also on Bruchomyiinae.

Description: Body length: 1.72 mm; wing: length 2.10 mm, width 0.71 mm.

Head: with a crest of elongate setae inserted on vertex and frons; cervical sclerites elongate, longer than width of eyes in lateral view; antennae with elongate-cylindrical flagellomeres decreasing in length (length in mm: 1/0.27; 2/0.18), each constricted at base and apex, covered with dense setae, shorter than those on other body parts, scapus 0.04 mm long, pedicel 0.07 mm long; labellum elongated; palps elongate, five-segmented (length in mm: 2/0.14; 3/0.12; 4/0.16; 5/0.36), basal segment inconspicuous (not possible to measure), ultimate segment strongly elongated, slightly longer than two previous ones combined (Figure 1B).

Thorax: wing is clearly visible; Sc ends almost opposite ¾ of length of R_4+5_ and slightly before cross-vein r-m; Rs ¹/₃ as long as R_4+5_ and is equal to e length of R_2_; R_2+3_ long, about 3 times longer than R_2_; cross-vein r-m 3 times shorter than M_1+2_; M_1_ slightly more than 4 times longer than M_1+2_; M_3_ slightly more than 8 times longer than M_3+4_; cross-vein m-m absent; cross-vein m-cu long, slightly longer than length of cross-vein r-m and located just before fork of M_3+4_ into M_3_ and M_4_; anal lobe reduced (Figure 1D). 

Legs with fringe of setae, most conspicuous at the joint between tibia and tarsi.

Abdomen: in general, typical of Bruchomyiinae; male genitalia elongate, more than three times longer than wide; gonocoxites about 0.18 mm long separate from each other at the base, with translucent membrane in between; gonostylus about 0.13 mm long, in almost ⅔ of basic length strongly extended, the end part of the gonostylus strongly narrowed and slightly sickle-shaped; aedeagus long, slightly curved, with apex bifurcate; parameters strongly curved, only slightly shorter than aedeagus (Figure 1A,C).

Material examined. Holotype male, No. MP/4044 deposited in the collection of the ISEA PAS. Myanmar amber, earliest Cenomanian (about 99 Ma).

Additional materials. MP/3704 (male; syninclusions: Araneae and Diptera), MP/3722 (male), MP/4044 (male), MP/4045 (male),MP/4046(male), MP/4047 (male) MP/4066 (male) all deposited in the ISEA PAS; NIGP 174740 (two males), NIGP 174741 (one male) deposited in the NIGP. 

Female unknown.

*Palaeoglaesum teres* sp. nov. Skibińska and Albrycht.

LSID urn:lsid:zoobank.org:act:C5BC6C53-721E-4DCC-BA99-65B38FB5D1E3.

**Figure 2:**
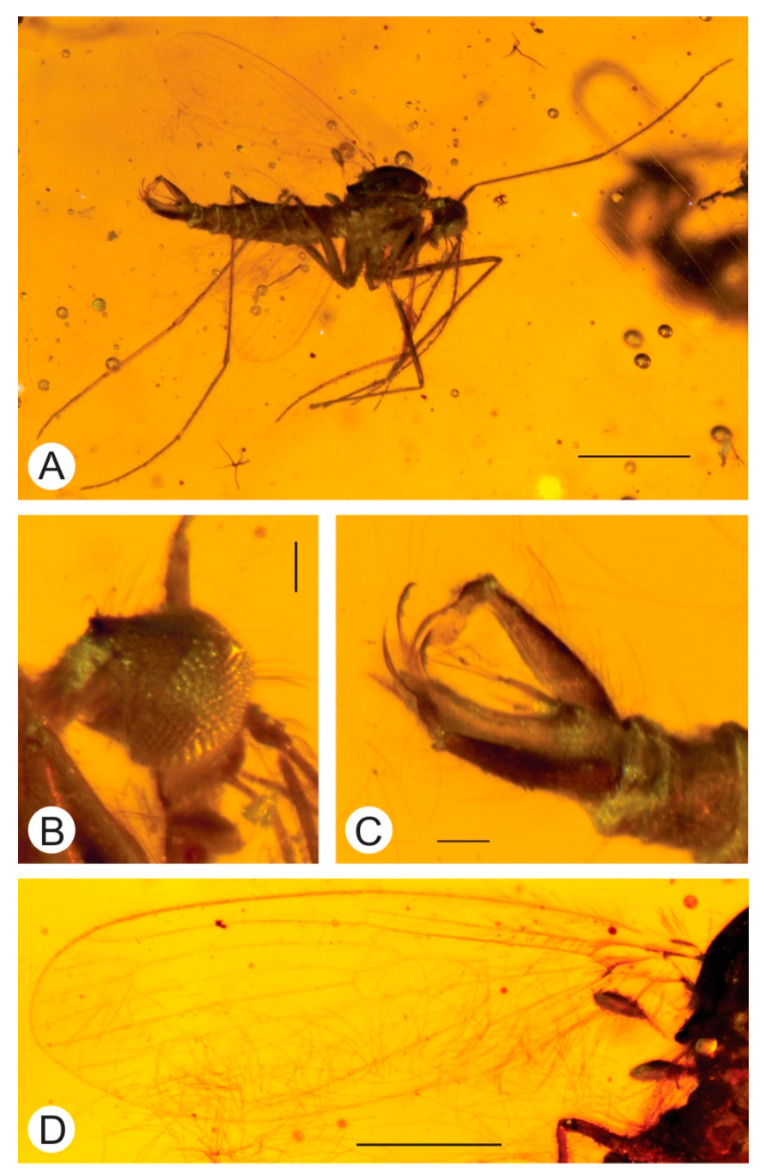
*Palaeoglaesum teres sp. nov.* Photos of the holotype [No. MP/3677]. (**A**) Whole specimen, scale: 1 mm; (**B**) head, scale: 0.1 mm; (**C**) male terminalia, scale: 0.1 mm; (**D**) wing, scale: 0.5 mm.

Diagnosis. Male with the following combination of character states: *Wing*: Sc ends just behind the fork of R_4+5_ into R_4_ and R_5_ and almost opposite cross-vein r-m; Rs is twice as long as R_4+5_ and is slightly shorter than the length of R_2_; M_1_ slightly more than 5 times longer than M_1+2_; *Male genitalia*: small, gonostylus forked, strongly expanded at the basal part; upper part of gonostylus narrow, slightly sickle-shaped, lower part clearly extended at ⅔ of its length, strongly tapered and curved at the end; aedeagus strongly curved and bifurcate in apical half; parameres short, strongly sickle-shaped, reaching about half of the aedeagus length (Figure 2).

Etymology. The new species is named after the first king of the Odrysian kingdom of Thrace, known mainly for his military skills.

Description: Specimen is well preserved. Body length: 2.2 mm; Wing length 1.9 mm, width 0.73 mm covered with sparse, long setae.

Head: with crest of elongate setae inserted on vertex and frons; antennae (boundaries between segments difficult to observe due to elongate setae) with narrow, elongate-cylindrical flagellomeres; first flagellomere about ⅓ longer than the next one and subsequent segments progressively shorter; palps elongate (not possible to measure), narrow, with four segments, the last segment is more than half as long as the penultimate one (Figure 2A,B).

Thorax: wing venation clearly visible; Sc ends just behind the fork of R_4+5_ into R_4_ and R_5_ and almost opposite cross-vein r-m; Rs twice as long as R_4+5_ and slightly shorter than length of R_2_; R_2+3_ about 2½ times longer than R_2_; cross-vein r-m about 2 times shorter than M_1+2_; M_1_ slightly more than 5 times longer than M_1+2_; M_3_ slightly more than 11 times longer than M_3+4_; cross-vein m-m absent; cross-vein m-cu about half the length of cross-vein r-m and located about its length behind fork of M_3+4_ into M_3_ and M_4_; anal lobe reduced (Figure 2D). 

Male genitalia: gonocoxite about 0.32 mm long; gonostylus about 0.22 mm long, forked, strongly expanded at basal part; upper part of gonostylus narrow, slightly sickle-shaped bent, lower part clearly extended at ⅔ of its length and strongly tapered and bent at the end; aedeagus strongly bent and bifurcate in apical half; short parameres, strongly sickle-shaped bent, reaching about half of aedeagus length (Figure 2C and Figure 5B).

Material examined. Holotype male, No. MP/3677 deposited in the collection of the ISEA PAS. Myanmar amber, earliest Cenomanian (about 99 Ma). Syninclusions: one Atelestidae, two Hymenoptera, one Brachycera, two Chironomidae, one Psocoptera, one Araneae, one Blattodea.

Female unknown. 

Remarks. This species is described based on a single specimen, which suggests that its presence in the fauna was scarce when Myanmar amber was forming. *Palaeoglaesum teres* sp. nov. is slightly similar to *P. wagneri* [5], however, it clearly differs in gonocoxite structure, sickle-like parameres, and the shape of the aedeagus, which is forked for almost half of its length.

*Palaeoglaesum pilosus* sp. nov. Skibińska, Krzemiński and Zhang.

LSID urn:lsid:zoobank.org:act:5DA7219F-01B4-490D-AD17-B0041D02A717.

**Figure 3:**
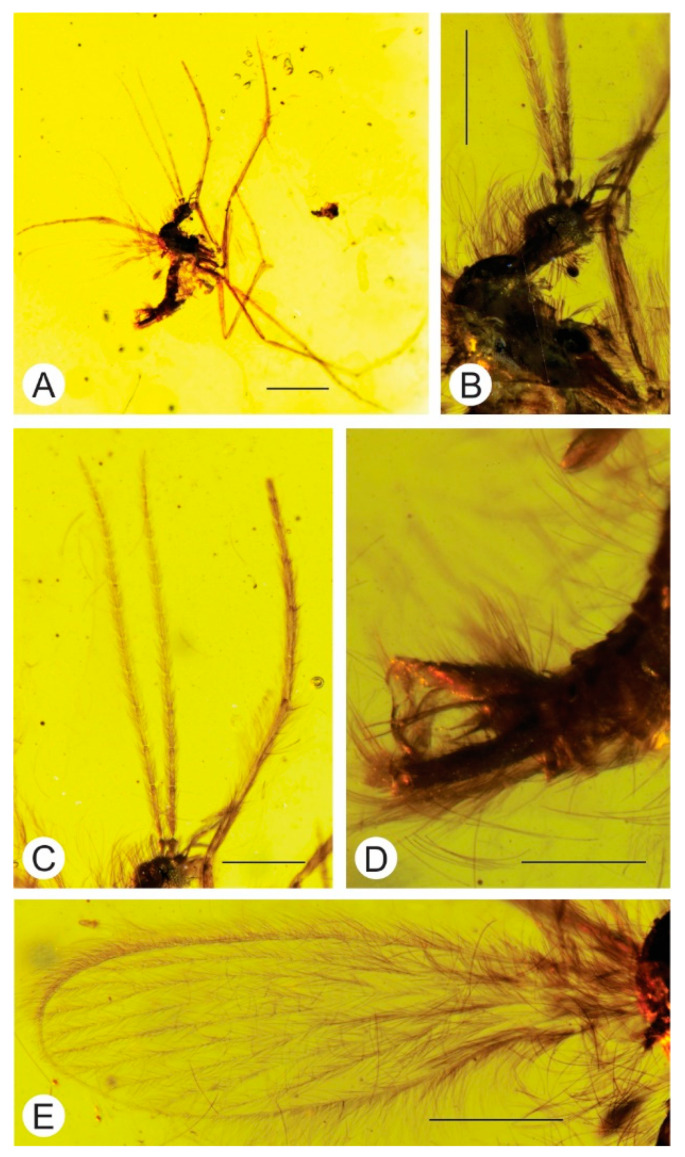
*Palaeoglaesum pilosus* sp. nov. Photos of the holotype [NIGP 174742]. (**A**) Whole specimen, scale: 1 mm; (**B**) head, scale: 0.5 mm; (**C**) antennae, scale: 0.5 mm; (**D**) male terminalia, scale: 0.5 mm; (**E**) wing, scale: 0.5 mm.

Diagnosis. Male with the following combination of character states: *Wing*: Sc ends almost opposite ¼ of the length of R_4+5_ and clearly before cross-vein r-m; Rs is ¹/₃ as long as R_4+5_ and is slightly longer than the length of R_2_; M_1_ slightly more than 9 times longer than M_1+2_. *Male genitalia*: gonostylus with a large appendix on the external edge, strongly expanded in a mid-length and tapered at the end; aedeagus strongly curved and bifurcated at about ¹/₃ of its length; short, sickle-shaped parameres, reach about ¹/₃ of aedeagus length (Figure 3).

Etymology. The specific epithet is in reference to the characteristic hairiness of the body which is typical for the whole genus.

Description. Head, thorax, and legs covered with long and dense setae. Body length about 1.83 mm; wing length about 2.18 mm; wing width about 0.7 mm (Figure 3).

Head: with crest of elongate setae inserted on vertex and frons; cervical sclerites elongate, longer than width of eyes in lateral view; antennae with elongated-cylindrical flagellomeres decreasing in length (length in mm: 1/0.31; 2/0.23) each constricted at base and apex, covered with dense setae, shorter than those on other parts of body, scapus 0.05 mm long, pedicel 0.08 mm long; labellum elongated; palps elongate, with four segments (not possible to measure) (Figure 3A,B).

**Figure 4:**
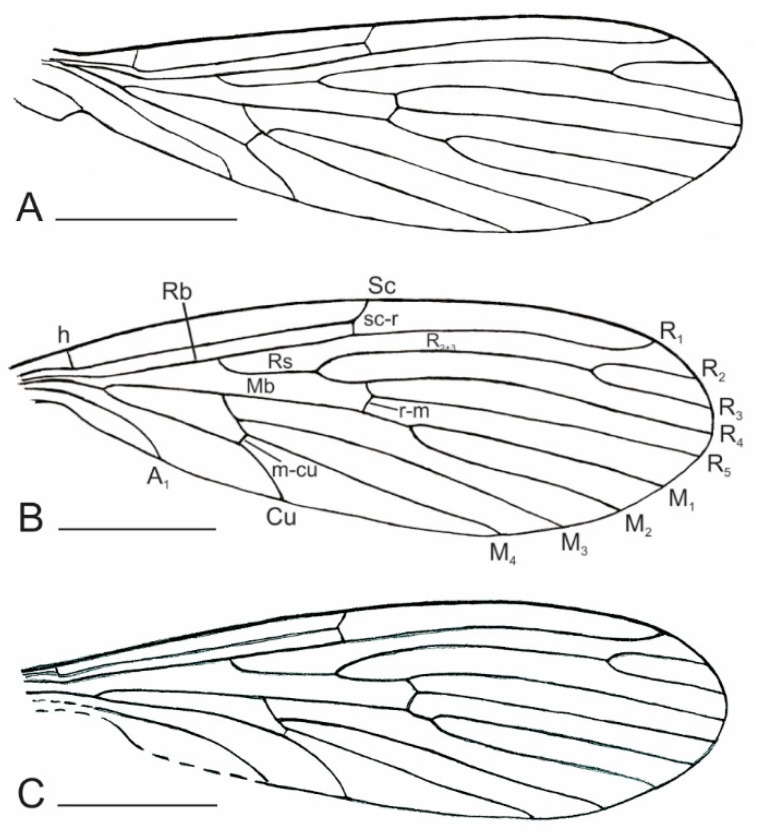
Line drawings of wing venation of (**A**) *P. stebneri* sp. nov.; (**B**) *P. teres* sp. nov.; (**C**) *P. pilosus* sp. nov. Scales: 0.5 mm. Abbreviations: A_1_ = first anal vein; Cu =cubital vein; M_1_ = first branch of media; M_2_ = second branch of media; M_3_ = third branch of media; M_4_ = fourth branch of media; R_1_ = anterior branch of radius; R_2_ = upper branch of second branch of radius; R_3_ = lower branch of second branch of radius; R_4_ = upper branch of third branch of radius; R_5_ = lower branch of third branch of radius; Sc = subcostal vein.

Thorax: wing venation clearly visible; Sc ends almost opposite **¼** of the length of R_4+5_ and clearly before cross-vein r-m; Rs is ¹/₃ as long as R_4+5_ and is slightly longer than the length of R_2_; R_2+3_ about 2¾ times longer than R_2_; cross-vein r-m is about the same length as M_1+2_; M_1_ slightly more than 9 times longer than M_1+2_; M_3_ almost 12 times longer than M_3+4_; cross-vein m-m absent; cross-vein m-cu short, twice as short as the length of cross-vein r-m and is located slightly further than its length behind the fork of M_3+4_ into M_3_ and M_4_; anal lobe reduced (Figure 3E and Figure 4C). 

Legs: covered with fringe of setae, most conspicuous at the joint between tibia and tarsi.

**Figure 5:**
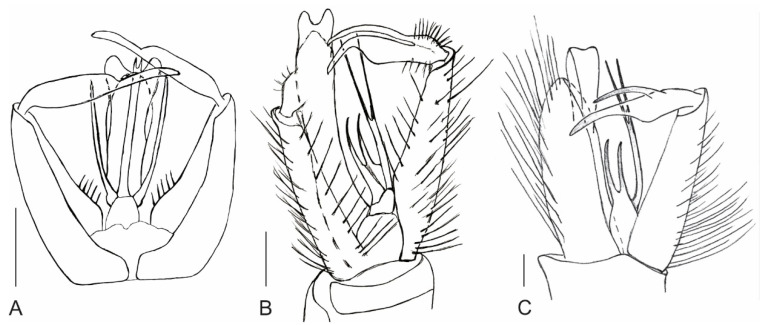
Line drawings of male genitalia of (**A**) *P. stebneri* sp. nov.; (**B**) *P. teres* sp. nov.; (**C**) *P. pilosus* sp. nov., scales: 0.1 mm.

Male genitalia: gonocoxite wide at a base, about 0.27 mm long; gonostylus about 0.20 mm long with a large appendix on the external edge, strongly extended at mid-length and visibly tapering to a point; aedeagus strongly curved and bifurcated at about ¹/₃ of its length; short, sickle-shaped parameres, reach about ¹/₃ of the aedeagus length (Figure 3C and Figure 5C).

Material examined. Holotype male, NIGP 174742 deposited in the collection of NIGP. Myanmar amber, earliest Cenomanian (about 99 Ma).

Additional materials. NIGP 174743 male, NIGP 174744 male deposited in the collection of NIGP, and MP/4047 male deposited in the collection of ISEA PAS. 

Female unknown. 

## 4. Discussion

Currently, Myanmar amber is of great interest among researchers. Its popularity is the effect of a huge variety of inclusions, their relatively high repeatability, and good state of preservation. Additionally, we find there both representatives of extinct evolutionary lines as well as the first evidence of the presence of contemporary Diptera groups. The first fossil Psychodidae were reported from the early Jurassic [17], and therefore they constitute one of the oldest clades of Diptera. Representatives of this family are found in subsequent geological periods, including all types of fossil resin, but their diversity and repeatability is considered to be the most numerous in Cretaceous Myanmar amber. In this fossil resin, significant differences between species and great diversity can also be observed within this ancient family, indicating its rapid evolutionary development during the Cretaceous period. Only from this fossil resin representatives of the extinct subfamily Datziinae [8] or the unique fossil Horaiellinae [18] were reported. Unfortunately, despite the huge number of inclusions available for research, so far no inclusions of larvae or pupae of genus *Palaeoglaesum* have been found and the larval habitat remains unknown, and it requires more materials to solve this issue. However, it seems that in the areas of amber resin production there were relatively few typically aquatic environments, but there could have been more environments with shallow, periodic water reservoirs and places with damp soil. Description of Bruchomyiinae larva which lives in forest litter, caves and moist, decomposed organic material [19] suggesting that *Palaeoglaesum* does not needs water reservoirs for its growth and that dark and damp places were enough. 

Our study confirms that *Palaeoglaesum* is the most species-rich genus of Bruchomyiinae found in the Myanmar amber. Currently, including the three new species described herein, a total of 10 are now recorded. Interestingly, such a well-represented and so highly diversified genus is known only from Myanmar amber and has not been described so far from other Cretaceous fossil resins such as Spanish or French amber. Consecutive described species indicate that contemporary representatives of the subfamily Bruchomyiinae should be treated as a relict group, which was far more widespread and diverse through the Mesozoic. However, it is highly plausible that its abundance was already significantly reduced in the Eocene, as only 13 species have been described from Baltic amber [20]. Moreover, it seems that this subfamily represented mainly by the genus *Palaeoglaesum* was dominant during this period (personal observation). However, it is interesting that subfamilies which are very common in contemporary fauna are relatively scarce (Trichomyinae) or not present (Psychodinae) in Myanmar amber [21].

## 5. Conclusions

New fossil materials show that during the Cretaceous period Bruchomyiinae live through very rapid radiation which resulted in the creation of many new species, moreover it seems that in this subfamily the genus *Palaeoglaesum* was one of the most widespread genera and could be easily distinguished by its small size and specific male genitalia with a sickle-bent, bifurcated aedeagus. 

Up until now, there have been no descriptions of larvae or pupas of this genus so the larval habitat of *Palaeoglaesum* remains unknown. However, the remarkably rich *Palaeoglaesum* inclusions in Myanmar amber may suggest that an environment with shallow, periodic water reservoirs and many places with damp soil were ideal for the development of these flies. 

## References

[1] Alexander C.P. (1920). A new subfamily of Tanyderidae flies (Diptera). Ann. Entomol. Soc. Am..

[2] Edwards F.W. (1921). A note on the subfamily Bruchomyiinae (Diptera Nematocera). Ann. Mag. Nat. Hist..

[3] Wagner R., Stuckenberg B.R. (2016). Cladistic analysis of subfamily Bruchomyiinae (Diptera: Psychodidae). Zootaxa.

[4] Fairchild G.B. (1952). Notes on *Bruchomyia* and *Nemopalpus* (Diptera, Psychodidae). Ann. Entomol. Soc. Am..

[5] Skibińska K., Krzemiński W., Zhang Q. (2019). A revised diagnosis of *Palaeoglaesum* Wagner (Diptera, Psychodidae, Bruchomyiinae) with description of two new species from Cretaceous Myanmar amber. Hist. Biol..

[6] Wagner R. (2017). Synopsis of extinct Bruchomyiinae (Diptera, Psychodidae) from Burmese, Baltic and Dominican amber, with description of a new genera and species. Zootaxa.

[7] Wagner R., Stuckenberg B.R. (2012). New fossil and extant species of *Nemopalpus* Macquart (Diptera: Psychodidae, Bruchomyiinae). S. Afr. Invertebr..

[8] Stebner F., Solórzano-Kraemer M.M., Ibáñez-Bernal S., Wagner R. (2015). Moth flies and sand flies (Diptera: Psychodidae) in Cretaceous Burmese amber. PeerJ.

[9] Byers G.W. (1989). Homologies in wing venation of primitive Diptera and Mecoptera. Proc. Entomol. Soc. Wash..

[10] Krzemiński W., Krzemińska E. (2003). Triassic Diptera: Descriptions, revisions and phylogenetic relations. Acta Zool. Crac..

[11] Cumming J.M., Wood D.M., Kirk-Spriggs A., Sinclair B. (2017). Adult morphology and terminology. Manual of Afrotropical Diptera.

[12] Cruickshank R.D., Ko K. (2003). Geology of an amber locality in the Hukawng Valley, northern Myanmar. J. Asian Earth Sci..

[13] Grimaldi D.A., Engel M.S., Nascimbene P.C. (2002). Fossiliferous Cretaceous amber from Myanmar (Burma): Its rediscovery, biotic diversity, and paleontological significance. Am. Mus. Novit..

[14] Shi G., Grimaldi D.A., Ge H., Wang J., Yang M., Lei W., Li Q., Li X. (2012). Age constraint on Myanmar amber based on U-Pb dating of zircons. Cretac. Res..

[15] Linnaeus C. (1758). Systema Naturae per Regna Tria Naturae, Secundum Classes, Ordines, Genera, Species, cum Caracteribus, Differentiis, Synonymis, Locis.

[16] Newman E. (1834). Attempted division of British insects into natural orders. Entomol. Mag..

[17] Ansorge J. (1994). Tanyderidae and Psychodidae (Insecta: Diptera) from the lower Jurassic of northeastern Germany. Palaeontol. Z..

[18] Curler G.R., Krzemiński W., Skibińska K. (2019). The first record of fossil Horaiellinae (Diptera: Psychodidae) from mid-Cretaceous amber of northern Myanmar. Cretac. Res..

[19] Hanson W.J. (1968). The Immature Stages of the Subfamily Phlebotominae in Panama Diptera: Psychodidae). Ph.D. Thesis.

[20] Curler G.R., Skibińska K. (2021). Paleotelmatoscopus, a proposed new genus for some fossil moth flies (Diptera, Psychodidae, Psychodinae) in Eocene Baltic amber, with description of a new species. Zootaxa.

[21] Ross A.J. (2020). Burmese (Myanmar) Amber Taxa, On-line Supplement v.2020.1. http://www.nms.ac.uk/explore/stories/natural-world/burmese-amber/.

